# Bacterial infections in a pediatric cohort of primary and acquired complement deficiencies

**DOI:** 10.1186/s12969-020-00467-0

**Published:** 2020-09-24

**Authors:** Taha Al-Shaikhly, Kristen Hayward, Matthew L. Basiaga, Eric J. Allenspach

**Affiliations:** 1 Division of Pulmonary, Allergy & Critical Care Medicine, Department of Medicine, Penn State College of Medicine, Hershey, PA USA; 2 Division of Rheumatology, Department of Pediatrics, University of Washington, Seattle, WA 98101 USA; 3 Division of Pediatric Rheumatology, Department of Pediatric and Adolescent Medicine, Mayo Clinic, Rochester, MN USA

**Keywords:** Systemic lupus Erythematosus, SLE, Connective tissue disease, Complement

## Abstract

**Background:**

Acquired complement deficiency can occur in the setting of autoimmune syndromes, such as systemic lupus erythematosus (SLE), with very low or, occasionally, undetectable C3 levels. Based on inherited complement defects, patients with transiently low complement may be at similar risk for serious bacterial infection, but the degree of risk related to C3 level and temporal association is unknown.

**Methods:**

We performed a retrospective study including pediatric patients with undetectable total complement activity or absent individual complement components measured at our institution from 2002 to 2018. We assessed annual rate of serious bacterial infection (SBI) defined as requiring hospitalization and/or parenteral antibiotics by manual chart review. Among included SLE patients, we assessed the 30-day probability of SBI for given C3 measurements using a logistic regression model to determine risk. Primary complement deficiency was analyzed for SBI rate as comparison. Covariates included age, level of immune suppression and history of lupus nephritis.

**Results:**

Acquired complement deficiency secondary to SLE-related disease [*n* = 44] was the most common underlying diagnosis associated with depressed complement levels and were compared to a cohort of primary complement deficient patients [*n* = 18]. SBI per 100 person-years and cohort demographics were described in parallel. Our logistic regression analysis of pediatric patients with SLE showed low C3 level was temporally associated with having an SBI event. Given equivalent immunosuppression, patients with an SBI had lower C3 levels at the beginning of the observation period relative to patients without SBI.

**Conclusion:**

Pediatric patients with the diagnosis of SLE can develop very low C3 levels that associate with risk of serious bacterial infection comparable to that of patients with primary complement deficiency. Patients prone to severe complement consumption may particularly be at risk.

## Background

Primary complement deficiencies are rare genetic conditions associated with clear susceptibility to encapsulated bacterial infections [1]. Secondary complement defects from an acquired disease process, such as systemic lupus erythematosus (SLE) or connective tissue disease (CTD), are usually partial and transient from immune complex formation and deposition. Temporary hypocomplementemia has been proposed to be a significant risk of infection among patients with SLE [2, 3]. Patients with SLE rarely demonstrate undetectable complement activity and roughly half of patients with active lupus have normal complement levels [4]. With infections still ranking as one of the highest causes for mortality in patients with SLE, understanding the associated risk factors is critical [5–7].

In this study, we utilized a large catchment area as one of the sole centers with combined Pediatric Rheumatology and Immunology expertise in the greater Pacific Northwest. We report serious bacterial infections (SBI) among patients with SLE/CTD-related disorders with a history of complement consumption, including detailed clinical history to account for kidney disease, immune suppression and types of infection, compared to that of an established cohort of primary complement deficient patients.

## Methods

Seattle Children’s Hospital institutional review board waived consent due to the study not involving greater than minimal risk (IRB Approval STUDY00001067). A convenience cohort was identified through retrospective review of institutional electronic medical records. Pediatric patients tested for complement activity between 2002 to 2018 were screened. Included subjects had undetectable total complement activity (CH50 or AH50) or undetectable individual complement component(s) at any timepoint. C3 and C4 complement levels were tested by nephelometry method performed at Seattle Children’s Hospital. CH50 and AH50 testing were performed by hemolytic methodology, while individual complement components and function were performed at National Jewish Health Complement Laboratory. Charts were manually reviewed to extract patient gender, date of birth, diagnosis, number and date of infections, immunosuppressive medications, treatment setting (inpatient versus outpatient), underlying microorganism(s), and any receipt of parenteral antibiotics and all complement measurements. Patients were categorized as primary complement deficiency (PCD) based upon clinical diagnosis, family history and/or individual factor deficiency. Period of observation started at birth until 2018 or lost to follow up in PCD and from diagnosis for secondary complement deficiency. Infections requiring hospitalization or parenteral antibiotics were included; 48-h empiric antibiotic treatments were excluded. Minor sinopulmonary infections, urinary tract infections or common skin infections not requiring parenteral antibiotics, and 48-h antibiotic treatments were excluded to limit analysis to clear life-threatening infectious risk. Serum samples drawn on the same day as SBI diagnosis were excluded. Highest levels of immunosuppression (LOI) were categorized by expert experience: Level 0, hydroxychloroquine only; Level 1, oral prednisone or methotrexate; Level 2, azathioprine or mycophenolate; Level 3, pulsed methylprednisolone and Level 4, cyclophosphamide, tacrolimus or rituximab [8]. Categories were not exclusive and higher LOI may also contain medications from lower LOI. Oral prednisone dose and duration were not quantified due to lack of clarity in the medical records. Washington State Immunization Information System (IIS) database verified immunization status. Statistical analysis: Descriptive analyses were performed to determine the medians and interquartile ranges (IQR) for continuous variables, and numbers and percentages for categorical variables. Univariable logistic regression was performed to explore the association of demographic classification, underlying diagnosis, and C3 level on the likelihood of having an SBI. Multivariable logistic regression was performed to determine the odds of SBI in patients with a low C3 level compared to those with a normal C3 level. A second logistic regression model examined SBI and C3 level as a continuous variable. The predictions from this model were used to create a 30-day prediction for SBI based upon C3 level. All logistic regression models controlled for LOI, age and lupus nephritis diagnosis. A sensitivity analysis was performed with LOI defined as high or low/none. Statistical analysis was performed using STATA v16.0 (StataCorp, Texas, USA).

## Results

### Study participants

We performed an unbiased review of all complement levels measured at our hospital from 2002 to 2018 (*n* = 5876, from 1643 total patients). A total of 70 subjects met inclusion criteria, based on an undetectable complement measurement during the observation period. Both patients with primary complement deficiency (*n* = 18) and those with acquired complement deficiency (*n* = 52) were included in the cohort (Table 1). Patients clinically diagnosed with SLE or CTD were captured in our pediatric dataset as quarterly complement testing is usual standard practice at our center. Manual chart review identified total number of subjects with each diagnosis screened in our dataset. Primary complement deficiencies in this cohort included C1q deficiency (*n* = 3), C2 deficiency (*n* = 11), C6 deficiency (n = 3), and C8 deficiency (*n* = 1) (Table 2). Acquired complement deficiencies included SLE/CTD-related disorders (*n* = 45), which represented 27% of the SLE patients [*n* = 43 of 155], 11% of pediatric Sjögren’s [*n* = 1 of 9] and 3.7% of MCTD patients [*n* = 1 of 27]. Two patients had C3 and/or C5 nephritic factors and 5 patients had infection-related complement consumption. Median period of observation was 11.2 years (IQR, 5.25–17) for primary complement deficiency followed from date of birth and 4.33 years (IQR, 2.7–6.5) for secondary complement disorders followed from diagnosis. Demographic characteristics and diagnoses are summarized in Table 1.

**Table 1:** Demographic and Clinical Characteristics

Variable^a^	SLE/CTD	PCD
Total, n.	45	18 (C2 = 11, C6 = 3, C1 = 3, C8 = 1)
Female, n. (%)	42 (93)	5 (28)
Age (year)	14.7 (IQR: 12.8–16.4)	11.2 (IQR: 5.25–17)
Observation		
Period (year)	4.8 (IQR: 2.7–6.5)	11.2 (IQR: 5.25–17)
SLE + Nephritis, n	23	
SLE^c^, n	20	
Other CTDs, n	2	
LOI ^b^		
LOI 0 n. (%)	1 (2)	3 (16)
LOI 1 n. (%)	6 (13)	
LOI 2 n. (%)	10 (22)	
LOI 3 n. (%)	5 (11)	
LOI 4 n. (%)	23 (51)	
SBI, n	27	30
SBI per 100PY	12.3 (95%CI 8.6,17.4)	14.7 (95%CI 10.5,20.4)

*PCD* Primary complement deficiency, *SLE/CTD* Systemic lupus erythematosus/connective tissue disease (SLEwN: with nephritis, *SLE* without nephritis), *LOI* Level of immunosuppression

^a^ For interval variables, the medians and interquartile ranges are presented. For categorical variables, the total number and percentages are presented

^b^ LOI is the level of immunosuppression: 0: Hydroxychloroquine 1: Prednisone and/or methotrexate, 2: Azathioprine, and/or mycophenolate, 3: Methylprednisolone, 4: Rituximab, cyclophosphamide and/or tacrolimus. LOI reflects the highest level of immune suppression, thus subjects may also be taking medications listed for lower levels

^c^ SLE category without nephritis includes subjects with serositis, autoimmune cytopenias, arthritis, autoimmune hepatitis, and CNS involvement including anti-neuronal antibody+without MRI changes or CNS lupus with cerebellar involvement

**Table 2:** Clinical and laboratory characteristics of primary complement deficiency patients

Pt	Age^a^ (yrs)/Sex	Comp Def	Laboratory results	Infections history (SBI, n)
1	3 / M	C2	Undetectable CH50, ↓ C2 level and function; C1q, C1r, C1s levels normal	Pneumococcal pneumonia, *E. coli* bacteremia, otitis media; recurrent acute otitis media (SBI = 2)
2	12 / M	C6	Family history of C6 deficiency, undetectable CH50, ↓ C6 level; (C5, C7-C9 levels normal).	*Neisseria meningitidis* meningitis and bacteremia (SBI = 1)
3	4 / M	C2	Undetectable CH50, undetectable C2 level; (C1, C3- C9 levels normal)	Two episodes of Streptococcal bacteremia, one episode of streptococcal meningitis, recurrent sinusitis (SBI = 3)
4	15 / M	C6	Undetectable CH50, ↓ C6 level; (C3, C5, C7-C9 levels normal)	*Neisseria meningitidis* meningitis and bacteremia (SBI = 1)
5	11 / M	C1q	Undetectable CH50, ↓ C1 level and function, ↓ C1q, (C1r, C1s, C2-C9 levels and function normal)	*Streptococcal pneumoniae* bacteremia and meningitis (SBI = 1)
6	10 / F	C2	Undetectable CH50, undetectable C2 level; (C3-C9 levels normal)	*Neisseria meningitidis* meningitis, recurrent otitis media (SBI = 1)
7	21 / M	C8	↓ CH50, undetectable AH50, ↓ C8 level, (C2-C7, C9 levels, properdin level normal)	Two episodes of *Neisseria meningitidis* meningitis, recurrent otitis media (SBI = 2)
8	6 / M	C1q	Undetectable CH50, ↓ C1q level; (AH50, C1r, C1s, C2, C4 levels normal)	Streptococcal bacteremia (SBI = 1)
9^b^	5 / M	C2	Family history of C2 deficiency, undetectable CH50, ↓ C2 level	None (Sjogren’s syndrome) (SBI = 0)
10^b^	4 / F	C1q	Family history of C1 deficiency, undetectable CH50, low C1q level	None (Discoid lupus) (SBI = 0)
11	3 / M	C2	Undetectable CH50, ↓ C2 level and function	Group B streptococcal meningitis and bacteremia; *Streptococcal pneumoniae* bacteremia, otitis media (SBI = 2)
12	18 / F	C6	Family history of C6 deficiency, undetectable CH50 and AH50, undetectable C6 function	None (erythromelalgia) (SBI = 0)
13	20 / F	C2	Undetectable CH50, previous physician diagnosis.	Recurrent otitis media and sinusitis; recurrent UTI, pyelonephritis (SBI = 2)
14	18 / M	C2	Undetectable CH50, undetectable C2 level and function; (AH50, C1, C4 levels normal)	Recurrent otitis media and sinusitis; multifocal bacterial pneumonia, (SBI = 2)
15	17 / M	C2	Undetectable CH50, undetectable C2 level and function; (C1, C3-C9 levels normal)	*Streptococcus pneumoniae* meningitis (SBI = 2)
16	13 / M	C2	Undetectable CH50, undetectable C2 level and function; (C1, C3-C9 levels normal)	Streptococcal bacteremia, *Hemophilus influenza* pneumonia (SBI = 2)
17^b^	18 / F	C2	Undetectable CH50, undetectable C2 level and function; (C3, C4 levels normal)	*Neisseria meningitidis* septic arthritis, spontaneous bacterial peritonitis (SBI = 2)
18	5 / M	C2	Undetectable CH50, undetectable C2 level and function; (C1, C3, C4 levels normal)	Recurrent episodes of *Streptococcus pneumoniae* bacteremia (SBI = 6)

↓, low; NL, normal

^a^ Age is calculated at the time of last clinical evaluation

^b^ Patients on hydroxychloroquine

### Serious bacterial infections

To examine the risk of SBI among patients with acquired complement deficiency, we first established the rate of SBI in our cohort of primary complement deficiencies with a known risk for encapsulated bacterial infections: overall, 30 SBI were recorded; SBI per 100 patient-years (PY) was 14.7 (95% CI 10.5, 20.4). The observation period for the SLE/CTD cohort started with the lowest C3 recorded value, typically being initial diagnosis, and ended with the last clinical visit recorded. Overall, 27 SBIs were recorded in our SLE/CTD cohort with 14 of 43 SLE/CTD patients (32%) having at least one SBI event during their observation period including pneumonia (33%), bacteremia (22%), sepsis (11%), intraabdominal infection (11%) and soft tissue infections requiring parenteral antibiotics (7%). *Streptococcus pneumoniae* and *Staphylococcus aureus* were the most frequently isolated microorganisms. Equivalent rates of vaccination for PCV13 and MCV4 were observed in the subjects with or without SBI, as well as rates of PPVS23 (albeit only roughly 40% of the SLE/CTD patients were fully immunized). SLE/CTD patients had a lower incidence with 12.3 SBI per 100PY (95% CI 8.6, 17.4) following lowest C3 level, although severe hypocomplementemia was transient.

### Complement levels

A total of 1197 serum measurements were collected over the observation period for the SLE/CTD cohort with a median of 5.32 separate serum measurements (IQR 3.73–6.35) per year collected per patient with a vast majority of the serum measurements reporting both C3 and C4 levels. Patients with the diagnosis of SLE/CTD all had either an undetectable (89%) or low C4 level (range, 2–6 mg/dL) (11%), at some point during their disease course. C3 levels were normal in 9% (*n* = 4) of the SLE/CTD cohort. We found trough C3 levels were lower in patients with SBI (35 mg/dL +/− 21) compared with patients without SBI (55 mg/dL +/− 24) (*P* = 0.01). Isolating the patients with higher LOI (3 or 4), the C3 level at the beginning of observation was lower on average in those with SBI (*P* = 0.031). Isolating lupus nephritis patients, the C3 level at the beginning of observation trended lower in those with SBI compared to those without (*P* = 0.139) (Supplemental Figure 1). Similar to LN and general LOI category, a severely depressed C3 level could represent more active disease.

**Fig. 1:**
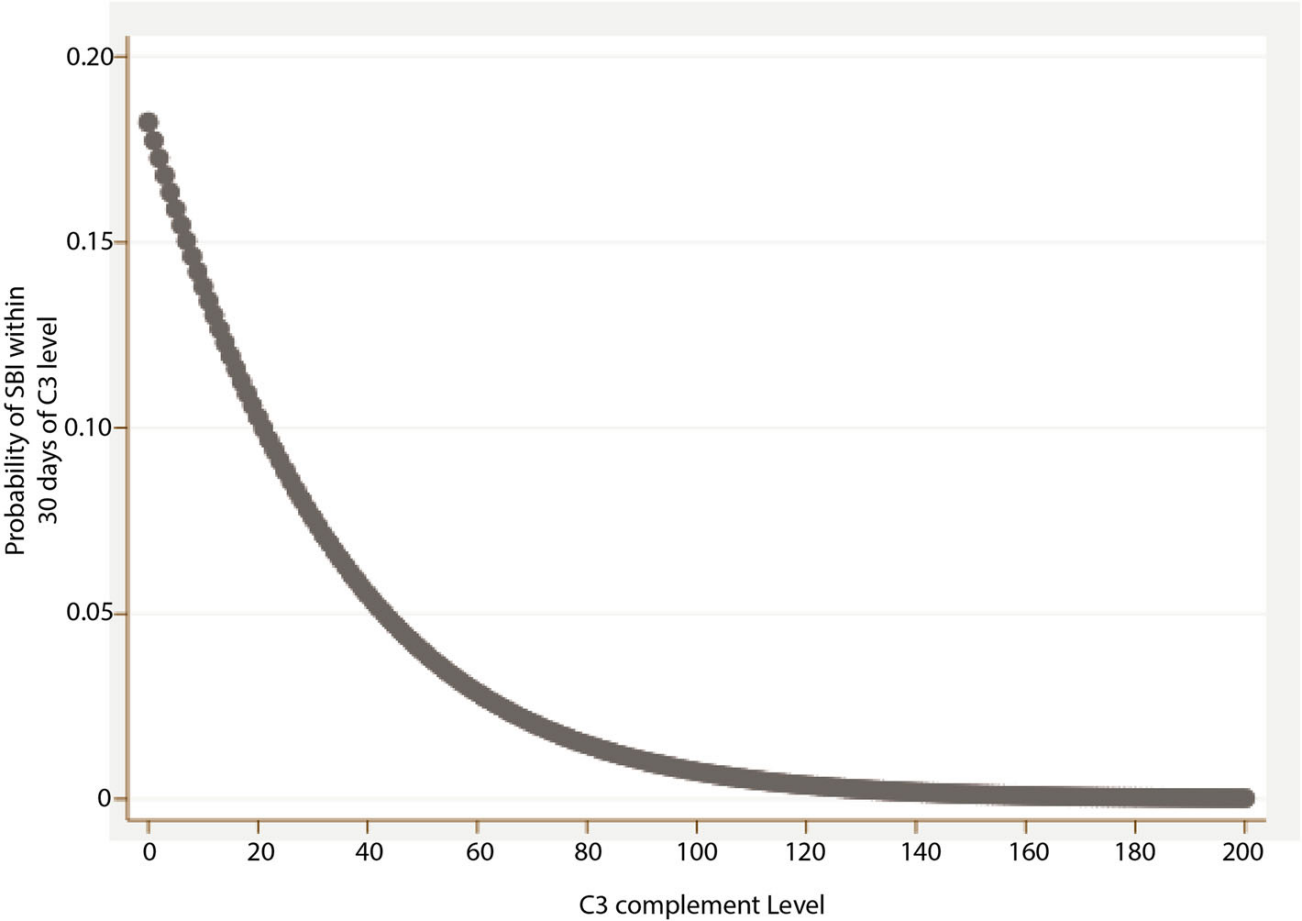
Logistic Regression analysis of C3 level in SLE/CTD patients. Logistic regression analysis examining C3 level as a predictor of the 30-day odds of having a serious bacterial infection adjusted for level of immunosuppression and lupus nephritis diagnosis in patients with SLE/CTD

To test whether low C3 levels were temporally associated with SBI in our cohort, we next utilized every C3 measurement for the SLE/CTD patients as a new observation (*n* = 1150) and assessed the 30-day probability of having an SBI based upon C3 level as a continuous variable (Fig. 1). We also categorically examined the risk of an infection in patients with low C3 (C3 < 83 mg/dL per reference range) compared to those with a normal C3 level (Table 3). Lupus nephritis and LOI have been associated with SBI in SLE cohorts, thus these were included as covariates. We excluded C3 levels drawn on the same day as the SBI diagnosis. In our cohort of SLE/CTD patients with a history of at least one measurement with undetectable complement, we found a correlation between a low C3 level and the probability of being diagnosed with an SBI within 30 days (Fig. 1). Further, our model suggests that patients with a very low to undetectable C3 level have an approximately 15% chance of being diagnosed with an SBI within 30 days. After controlling for age, level of immune suppression, and lupus nephritis diagnosis, logistic regression showed that patients with a low C3 measurement have a significantly higher risk of infection than those with a normal C3 level (OR 5.34 [95% CI, 1.88–15.16]) (Table 3). None of these covariates were themselves significantly associated with SBI in our model. Level of immune suppression had a trend towards significance, but our sample size limited our ability to develop an odds ratio for each respective LOI level. Given that infection itself may be a causal event for undetectable complement, a secondary analysis excluded any C3 levels drawn within 30 days following an SBI and we found similar results.

**Table 3:** Odds of Serious Bacterial Infection following C3 level in children with SLE/CTD diagnosis

Serious Bacterial Infection (SBI)	Odds Ratio	Std. Err.	Z	P > |z|	95% Conf. Interval
**C3 (Low)**	5.52	3.00	3.15	**0.002**	(1.90–16.00)
**LOI**	3.56	2.38	1.89	0.058	(0.96–13.23)
**LN Present**	1.52	0.83	0.77	0.77	(0.52–4.42)

Logistic regression analysis of serious bacterial infection within 30 days of a respective C3 level. Regression model accounts for level of immune suppression (LOI), history of lupus nephritis (LN), and age

## Discussion

Here, we demonstrate longitudinal infection data on two pediatric cohorts from our institution demonstrating comparable infection rates in primary and secondary complement deficiencies. Although clinicians generally appreciate that patients with low complement levels predispose for bacterial infections, the degree of complement depletion and duration of deficiency remains hard to quantify the risk. Our unique study design focused on SLE and CTD subjects prone to complement depression to demonstrate that a low C3 level is associated with a significant risk for serious bacterial infection within 30 days in pediatric SLE/CTD patients adjusting for general immunosuppression level and lupus nephritis diagnosis. Our findings are consistent with previous studies that found suboptimal C3 levels at time of SLE diagnosis or decreased total complement activity (CH50 < 30 U/mL) to be associated with higher infection risk [2, 9]. Increased infection risk among SLE patients in our cohort is likely multifactorial [10] and causation is not addressed.. Including complement levels in future prospective trials will be important to separate the individual risk components. Pediatric SLE/CTD with a tendency to consume complement may benefit from hyperimmunization and rapid receipt of empiric antibiotics similar to primary complement deficient patients [11]. Thus far, physicians are failing to recommend vaccines for SLE patients [12].

A major strength of the study is the large catchment area and lack of other local centers with expertise for these patients, making it less likely that referral bias has affected our results. It is routine care to screen patients with SLE and CTD at our institution with quarterly C3 and C4 complement levels at our center, which lessens the ascertainment bias. The study also benefited from the long observation period to provide a window into longitudinal risk. Being at single referral center permitted normalization of the testing and serum handling methods known to be critical in complement assessment. A sub-analysis excluding complement levels drawn shortly after an SBI did not affect our results, which supports the association of hypocomplementemia and SBI rather than hypocomplementemia being secondary to SBI. Lastly, we made efforts to normalize for confounding variables including immunosuppressive agents, lupus nephritis diagnosis and vaccination status of our cohort.

Our study has several limitations. Our retrospective analysis was limited by missing data, as some of the recorded infections lacked corresponding C3 levels in the preceding 30 days and no leukocyte counts were collected. Due to the lack of a well-established consensus, the grading of immunosuppression level in our cohort was based on expert clinical experience alone and lacked critical values known to be associated with SBI, namely duration and dose of steroids. Further prospective studies may be needed to control for these limitations and better examine the risk of infection in patients with complement defects due to C3 consumption as SLE patients are high risk for infections [13].

## Conclusion

Pediatric patients with severe C3 consumption represent a population with significant infectious risk comparable to that of patients with primary complement deficiency, even while adjusting for immunosuppression level and lupus nephritis diagnosis. Prospective validation is thus warranted to clarify the temporal risk and the level of complement depression associated with the risk in relation to immune suppression and disease activity.

## Supplementary information

**Additional file 1: Figure S1.** C3 level at the beginning of the period of observation compared based upon the presence or absence of a serious bacterial infection (SBI) within subject with a history of lupus nephritis (LN) (*left*) or level of immunosuppression (LOI) 3 or 4 (*right*).

## Data Availability

Not applicable.

## Abbreviations

SLE: Systemic lupus erythematosus
CTD: Connective tissue disease
SBI: Serious bacterial infection
LOI: Levels of immunosuppression
