# Supplementary material for: Bacterial infections in a pediatric cohort of primary and acquired complement deficiencies
Source: Pediatr Rheumatol Online J. 2020 Sep 24;18:74. doi: 10.1186/s12969-020-00467-0 (PMC7513499; doi:10.1186/s12969-020-00467-0)
Supplement: Supplementary file 1 — Additional file 1: Figure S1. C3 level at the beginning of the period of observation compared based upon the presence or absence of a serious bacterial infection (SBI) within subject with a history of lupus nephritis (LN) (left) or level of immunosuppression (LOI) 3 or 4 (right). [file 12969_2020_467_MOESM1_ESM.docx]

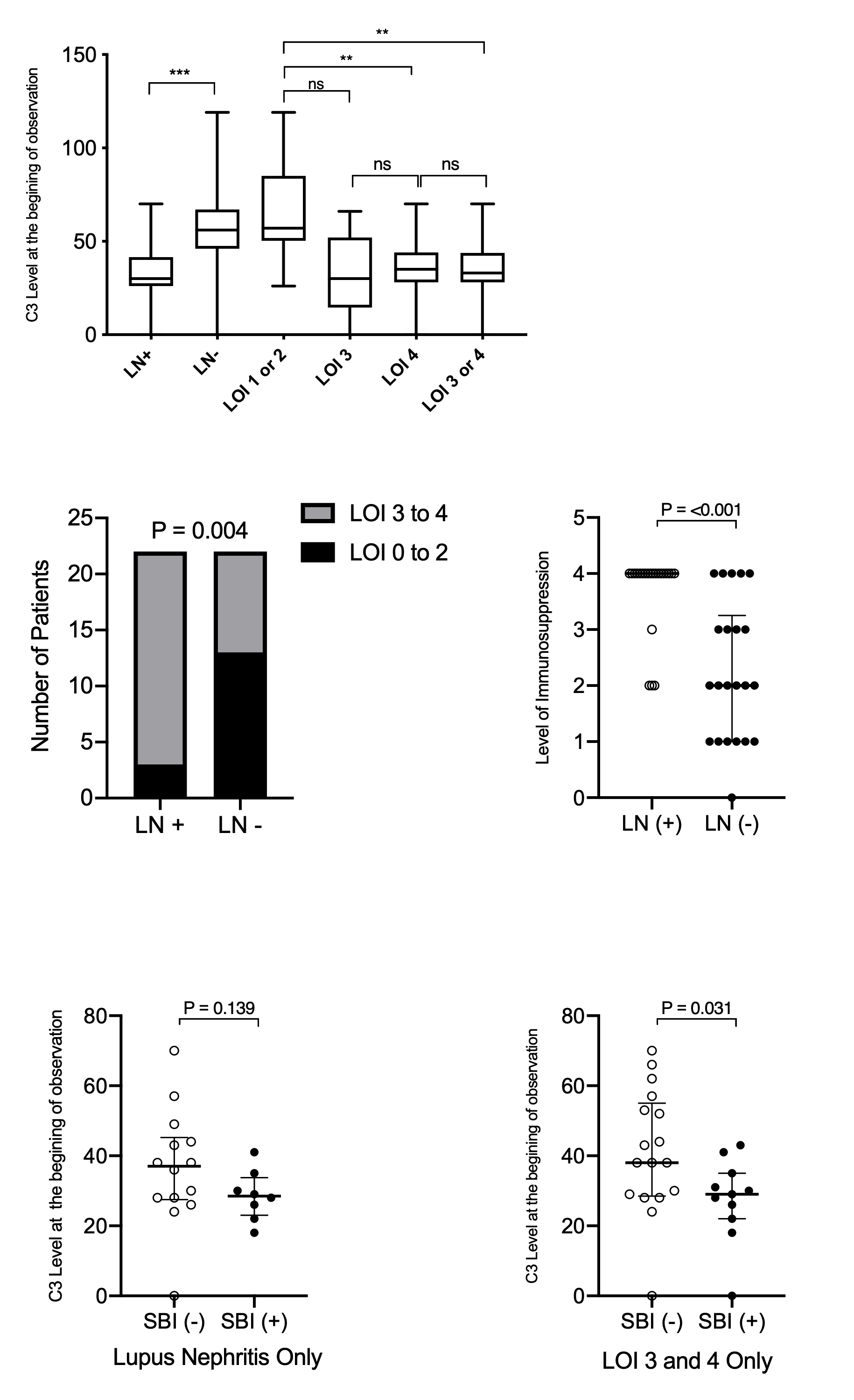


Supplemental Figure 1. C3 level at the beginning of the period of observation compared based upon the presence or absence of a serious bacterial infection (SBI) within subject with a history of lupus nephritis (LN) (*left*) or level of immunosuppression (LOI) 3 or 4 (*right*).
